# Molecular epidemiology and clinical‐laboratory aspects of chromoblastomycosis in Mato Grosso, Brazil

**DOI:** 10.1111/myc.13505

**Published:** 2022-09-29

**Authors:** Armando Guevara, Andréia Ferreira Nery, Márcia de Souza Carvalho Melhem, Lucas Bonfietti, Anderson Messias Rodrigues, Ferry Hagen, Jamile Ambrósio de Carvalho, Zoilo Pires de Camargo, Bruna Jacomel F. de Souza Lima, Vânia Aparecida Vicente, Rosane Christine Hahn

**Affiliations:** ^1^ Laboratory of Mycology/Research, Faculty of Medicine Federal University of Mato Grosso Cuiabá Mato Grosso Brazil; ^2^ Júlio Muller University Hospital Federal University of Mato Grosso Cuiabá Mato Grosso Brazil; ^3^ Health Department Mycology Nucleus of Instituto Adolfo Lutz São Paulo SP Brazil; ^4^ School of Medicine Federal University of Mato Grosso do Sul Campo Grande, MS Brazil; ^5^ Laboratory of Emerging Fungal Pathogens, Department of Microbiology, Immunology, and Parasitology, Discipline of Cellular Biology Federal University of São Paulo (UNIFESP) São Paulo Brazil; ^6^ Department of Medicine, Discipline of Infectious Diseases Federal University of São Paulo (UNIFESP) São Paulo Brazil; ^7^ Westerdijk Fungal Biodiversity Institute Utrecht The Netherlands; ^8^ Institute for Biodiversity and Ecosystem Dynamics (IBED) University of Amsterdam The Netherlands; ^9^ Department of Medical Microbiology Utrecht University Medical Center Utrecht The Netherlands; ^10^ Microbiology, Parasitology and Pathology Post‐Graduation Program, Department of Pathology Federal University of Paraná Curitiba Paraná Brazil

**Keywords:** AFLP, chromoblastomycosis, chromomycosis, epidemiology, *Fonsecaea pedrosoi*, molecular epidemiology

## Abstract

**Introduction:**

Chromoblastomycosis is a disease caused by melanized fungi, primarily belonging to the genera *Fonsecaea* and *Cladophialophora*, mainly affecting individuals who are occupationally exposed to soil and plant products. This research aimed to determine the clinical, epidemiological and laboratory characteristics of chromoblastomycosis in the state of Mato Grosso, Brazil.

**Materials and Methods:**

Patients diagnosed with chromoblastomycosis treated at the Júlio Müller University Hospital, Cuiabá, Brazil, from January 2015 to December 2020, whose isolates were preserved in the Research Laboratory of the Faculty of Medicine of the Federal University of Mato Grosso. Isolates were identified by partly sequencing the Internal Transcribed Spacer (ITS) and β‐tubulin (*BT2*) loci. AFLP fingerprinting was used to explore the genetic diversity. Susceptibility to itraconazole, voriconazole, 5‐fluorocytosine, terbinafine and amphotericin B was determined by the broth microdilution technique.

**Results:**

Ten patients were included, nine were male (mean age = 64.1 years). Mean disease duration was 8.6 years. Lesions were mainly observed in the lower limbs. Predominant clinical forms were verrucous and scarring. Systemic arterial hypertension and type II diabetes mellitus were the predominant comorbidities. Leprosy was the main concomitant infectious disease. *Fonsecaea pedrosoi* was the unique aetiological agent identified with moderate genetic diversity (*H* = 0.3934–0.4527; *PIC* = 0.3160–0.3502). Antifungal agents with the highest activity were terbinafine, voriconazole and itraconazole.

**Conclusion:**

Chromoblastomycosis is affecting the poor population in rural and urban areas, mainly related to agricultural activities, with *F. pedrosoi* being the dominant aetiologic agent. All isolates had low MICs for itraconazole, voriconazole and terbinafine, confirming their importance as therapeutic alternatives for chromoblastomycosis.

## 1 INTRODUCTION

Chromoblastomycosis is a chronic, transmissible disease endemic to tropical and subtropical regions, classified by the World Health Organisation (WHO) as a neglected tropical disease.^1^ It is caused by melanized fungi commonly present in soil, on plants and decaying wood. *Fonsecaea* and *Cladophialophora* are mainly involved, with *F. pedrosoi* and *C. carrionii* being the most frequent species. There are also case reports that reported other fungal species from the genera *Phialophora*, *Rhinocladiella*, *Exophiala*, *Cyphellophora*, *Veronaea*, *Phoma* and *Chaetomium*.^2^, ^3^, ^4^, ^5^, ^6^, ^7^

Chromoblastomycosis usually affects people who are occupationally exposed to plants and soil products, such as farmers, gardeners, loggers and sellers of agricultural products; therefore, it is considered an occupational disease.^4^, ^5^, ^8^, ^9^

Brazil is one of the countries most affected by chromoblastomycosis, with the Amazon region having the highest reported prevalence.^10^, ^11^ In Brazil, the actual disease incidence and prevalence are largely unknown, as it is not mandatory to notify this disease. However, there are reports of epidemiological and microbiological research that certify the presence of this disease in nearly the entire country,^7^, ^12^, ^13^, ^14^, ^15^, ^16^, ^17^, ^18^, ^19^, ^20^, ^21^ with an estimated prevalence of 0.098 cases per 1,000,000 inhabitants.^11^

In the scientific literature, there are few case reports of chromoblastomycosis from the state of Mato Grosso, in central‐western Brazil. This region has the conditions for the presence of chromoblastomycosis: humid tropical climate, diverse ecosystems (including part of the Amazon), extensive biodiversity and important agricultural and livestock production. This scenario has a large population of farmers exposed to the ecosystems where the aetiologic agents of chromoblastomycosis are present. Lack of knowledge of the clinical and epidemiological characteristics of patients in Mato Grosso and the aetiological agents of chromoblastomycosis in the state and their antifungal susceptibility profile makes it difficult to make correct therapeutic decisions that may benefit patients. Therefore, this research aimed to determine the clinical, epidemiological and laboratory characteristics of chromoblastomycosis in the Mato Grosso state, Brazil.

## 2 MATERIALS AND METHODS

We carried out a retrospective review of the medical records of chromoblastomycosis patients treated at the Infectious and Tropical Diseases Outpatient Clinic of the Júlio Müller University Hospital (JMUH), Cuiabá, Mato Grosso state, Brazil, from 1 January 2015, to 31 December 2020. Patients whose isolates were preserved in the Research Laboratory of the Faculty of Medicine of the Federal University of Mato Grosso (FM‐FUMT) were selected. The variables studied were as follows: age, sex, origin, occupation, location of lesions, history of trauma at the anatomical site of chromoblastomycosis lesions, number of affected body regions, evolution time of lesions from onset to disease diagnosis, types of lesions, symptoms, complications, laboratory diagnostic methods used (histopathology, direct microscopy, culture, molecular identification of isolates), identified aetiological agents, presence of immunosuppressive conditions and other concomitant infectious diseases. The types of lesions and disease severity were classified according to Queiroz‐Telles et al.^4^ According to severity, the lesions can be classified as mild if the patient has a solitary plaque or nodule less than 5 cm in diameter, moderate when the patient has a single or multiple nodule, plaque, or verrucous lesion covering one or two adjacent skin regions, measuring less than 15 cm in diameter and severe when the patient has any type of single or multiple lesion covering extensive skin regions, adjacent or not.^4^, ^5^ The diagnosis of chromoblastomycosis was confirmed by detecting muriform cells in the histopathological examination and/or direct examination of the samples and by the isolation and molecular identification of the aetiological agents.

### 2.1 Molecular identification of isolates

All aetiologic agents of chromoblastomycosis were identified by amplification and sequencing of the ITS (ribosomal DNA Internal Transcribed Spacer) and β‐tubulin (*BT2*) loci, according to previously published protocols.^22^, ^23^ The isolates were deposited at the ‘Microbiology Collection of the Taxonline Network—TAXonline at the Federal University of Paraná, in Parana state, Curitiba‐Brazil’ (http://repositorio.utfpr.edu.br/jspui/handle/1/3332). The DNA sequences were deposited in NCBI GenBank, and the accession numbers are listed in Table 2.

### 2.2 AFLP typing and bioinformatics analysis

AFLP typing was carried out following the protocol of Vos et al^24^ with minimal modifications as proposed by de Carvalho et al.^25^ Briefly, approximately 200 ng of *Fonsecaea* genomic DNA was in vitro digested using EcoRI (GˆAATTC) and MseI (TˆTAA) restriction enzymes (New England Biolabs, Ipswich, MA, USA) and simultaneously ligated to EcoRI and MseI adapters. EcoRI+0 and MseI+0 primers were employed for pre‐selective PCR.^24^ Fluorescent AFLP was performed with 6‐carboxyfluorescein (FAM) EcoRI primer with two bases selection (5′‐GAC TGC GTA CCA ATT CNN‐3′) and unlabelled MseI primer without bases selection (5′‐GAT GAG TCC TGA GTA A‐3′). We choose combinations #1 EcoRI‐AC/MseI‐0 and #2 EcoRI‐GA/MseI‐0 to screen genetic diversity among *Fonsecaea* isolates.

Amplicons were determined using capillary electrophoresis with a SeqStudio Genetic Analyser alongside a GeneScan LIZ600 internal size standard (35–600 bp; Applied Biosystems) under previously described conditions.^26^

Electropherograms were imported into BioNumerics v. 7.6 software (Applied Maths, Sint‐Martens‐Latem, Belgium), and distance‐based techniques were used to evaluate relationships among *Fonsecaea* isolates. A dendrogram demonstrating the genetic relationship among all *Fonsecaea* isolates was generated for each marker by applying the band‐based Jaccard similarity coefficient,^27^ the unweighted pair group mean arithmetic method (UPGMA) and the cophenetic correlation coefficient. The Pearson product–moment correlation coefficient (Pearson correlation)^28^ was used to assess the congruence between the two AFLP combinations.

The evolutionary relationships among all the genotypes of *Fonsecaea* isolates were investigated using principal component analysis (PCA), Self‐Organising Maps (SOM) and Minimum Spanning Trees (MSTs) as previously described.^29^, ^30^ The following descriptive genetic parameters for AFLP markers were calculated: polymorphic information content (*PIC*),^31^ expected heterozygosity (*H*),^32^ effective multiplex ratio (*E*),^33^ arithmetic mean heterozygosity (*Havp*),^33^ marker index (*MI*),^33^, ^34^ discriminating power (*D*)^35^ and resolving power (*Rp*).^36^ All figures were exported and treated using Corel Draw 2021.

### 2.3 Antifungal susceptibility

Antifungal susceptibility was determined by the broth microdilution method according to EUCAST definitive document E.DEF 9.3.2.^37^ The antifungals used were amphotericin B, voriconazole, itraconazole, 5‐fluorocytosine and terbinafine (Sigma‐Aldrich, St. Louis, MO, USA), provided as pure powders. The final concentration of the drugs ranged from 0.03 to 16 μg/ml diluted in RPMI1640 2% glucose medium buffered to pH 7.0 with morpholinopropane sulfonic acid. The suspensions of conidial inocula were prepared from 7‐ to 14‐day‐old cultures at 30°C on potato dextrose agar slants by addition of 5 ml sterile water with 0.1% Tween 20, followed by carefully rubbing the conidia with a sterile cotton swab. The suspension was adjusted with sterile distilled water to a final working inoculum density of 2–5 × 10^5^ cells/ml. The test incubation was done at 35°C, and the examination was carried out for 96–120 h according to the growth time of each strain. The MICs were determined by visual inspection of growth, and they corresponded to either prominent inhibition (50%) for 5‐fluorocytosine or complete growth inhibition (100%) for amphotericin B, terbinafine, itraconazole and voriconazole. The test was performed in duplicate, and the final MIC of each drug against each strain was obtained by geometric mean. *Candida krusei* ATCC 6258 (=CBS 573) and *C. parapsilosis* ATCC 22019 (=CBS 604) were used for test quality control.

### 2.4 Ethics aspects

The project was approved by the Research Ethics Committee of the JMUH under number: 3.733.631. The consultation of the medical records was duly authorised by the institution's clinical responsible. The epidemiological records of the patients involved in the study were coded with numbers to preserve their identity.

## 3 RESULTS

**FIGURE 1:**
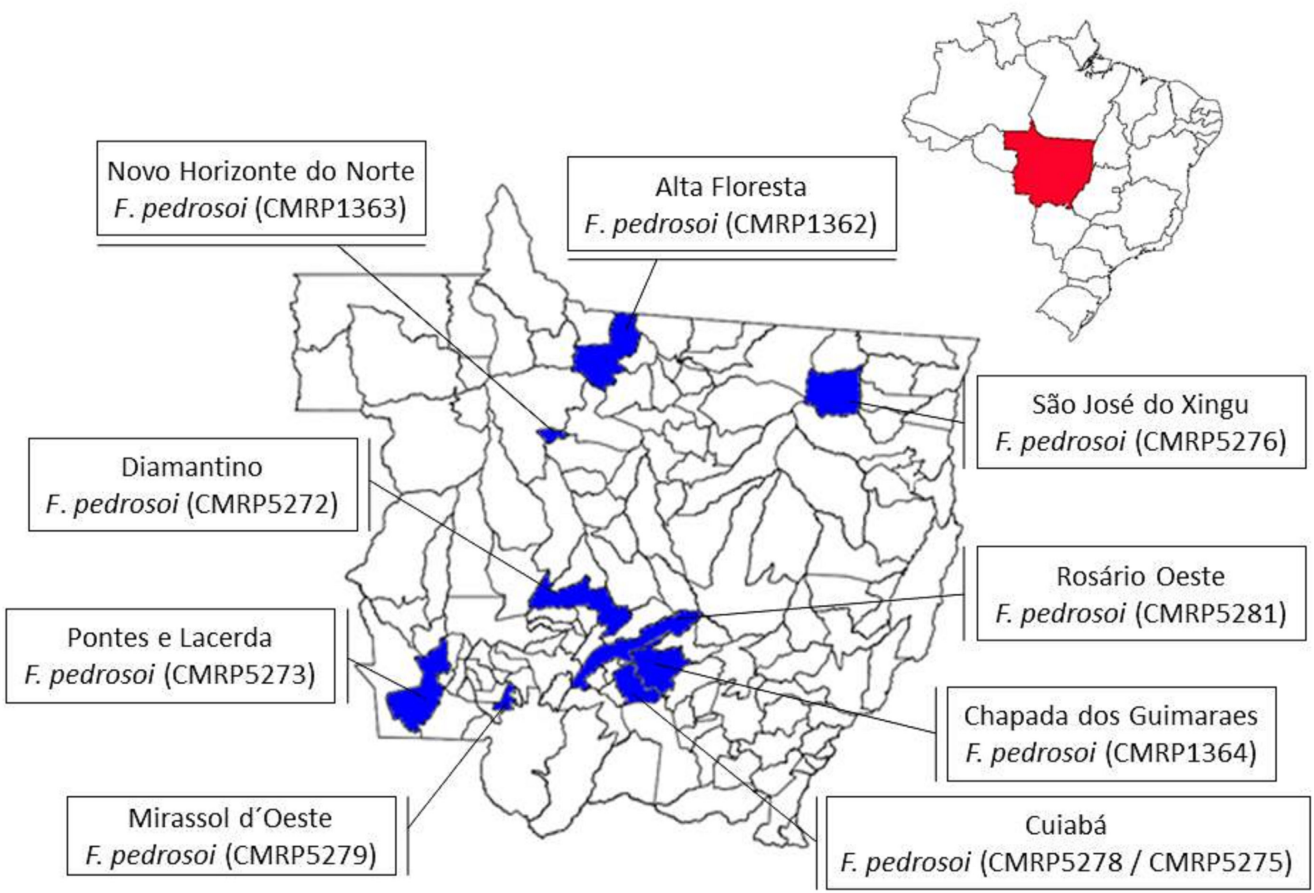
Municipalities in the Mato Grosso state, Brazil, and the species of chromoblastomycosis agents isolated

During the study period, 10 patients were identified from who their chromoblastomycosis‐causing isolates were preserved at the Research Laboratory at FM‐UFMT. The mean age was 64.1 years (49–91 years), nine of them were male (Table 1). Five patients resided in rural areas, and all reported the onset of the disease in the municipalities of origin (Figure 1). Occupations related to rural work predominated in this group of patients, with 6 farmers, 1 cattleman, 1 retired, 1 fumigator and 1 unemployed. The average time of disease evolution from the onset of the lesions to diagnosis was 102.6 months (8.6 years), ranging from 2 to 240 months. The patients 7, 8 and 9 were previously diagnosed with chromoblastomycosis at other healthcare centres. They were treated with different antifungal agents (patient 7: itraconazole, griseofulvin and terbinafine; patient 8: itraconazole; and patient 9: ketoconazole and itraconazole). It was unfortunately not possible to obtain the duration, doses and chronology of treatment data for these patients. Patient 7 also received 4 cryotherapy sessions, 3 of which were 12 years ago and 1 of them 5 years ago. The time of disease evolution in these patients was over 10 years. A patient with 12 years of evolution (patient 6) was initially diagnosed with paracoccidioidomycosis, treated with fluconazole (300 mg/day) for 4 years and then with itraconazole (200 mg/day) for 6 years without improvement of the lesions. None of these patients were under treatment at the time of the first consultation at the JMUH. A patient with 15‐year evolution of chromoblastomycosis had not been diagnosed earlier.

**TABLE 1:** Demographic and clinical characteristics of patients with chromoblastomycosis

Patient	Age (years)	Sex	Geographical origin	Occupation	Evolution (months)	Localization	Clinical features	Intensity	Symptoms	Complications	Comorbidities	Treatment/inicial outcome
1	60	M	Alta Floresta, MT, Brazil	Cattleman	48	Left knee	Verrucous	Mild	Pruritus	No	No	Itraconazole 200 mg/day for 4 year/ Improvement
2	76	M	Novo Horizonte do Norte, MT, Brazil	Farmer	48	Right forearm, right hand	Verrucous	Moderate	Pruritus	No	SAH, DM II	Itraconazole 200 mg/day for 5 year/ Improvement
3	60	M	Chapadas dos Guimaraes, MT, Brazil	Fumigator	4	Right leg	Verrucous	Mild	Foul odour	Bacterial infection	Hepatical cirrhosis, SAH, DM II	No antifungal treatment
4	49	M	Diamantino, MT, Brazil	Farmer	180	Left thigh, left leg	Mixed	Severe	Pruritus	Bacterial infection	No	Itraconazole 200 mg/day for 4 year, cryotherapy/ Improvement
5	91	F	Cuiabá, MT, Brazil	Retired	2	Left leg, left foot	Cicatricial	Moderate	Pruritus	No	SAH, osteoporosis, leprosy	Itraconazole 200 mg/day for 3 year, cryotherapy/ Improvement
6	57	M	Mirasol do Oeste, MT, Brazil	Farmer	144	Right leg, right foot	Verrucous	Severe	Pain, foul odour	Bacterial infection	Leprosy	Itraconazole 200 mg/day for 3 year, cryotherapy, sunflower oil/No improvement
7	57	M	Pontes e Lacerda, MT, Brazil	Farmer	180	Left thigh, knee, leg foot	Mixed	Severe	Pain, inflamation, foul odour	Bacterial infection	SAH, DM II	Itraconazole 400 mg/day for 3 year /No improvement
8	62	M	Cuiabá, MT, Brazil	Unemployed	240	Right arm, right forearm	Cicatricial	Severe	Pain	No	SAH, DM II	Itraconazole 400 mg/day for 4 year, cryotherapy/ Improvement
9	64	M	São José do Xingú, MT, Brazil	Farmer	120	Right thigh, right foot	Verrucous	Severe	Pruritus, pain	No	SAH	Itraconazole 400 mg/day for 2 year, cryotherapy/ Improvement
10	65	M	Rosario Oeste, MT, Brazil	Farmer	60	Left elbow	Cicatricial	Mild	No	No	COPD, leprosy, BPH, arterial insufficiency in RLL	No antifungal treatment

Abbreviations: BPH: Benign prostatic hyperplasia; COPD: Chronic obstructive pulmonary disease; DM II: Type II diabetes mellitus; MT: Mato Grosso state; RLL: Right lower limb; SAH: Systemic arterial hypertension.

None of the patients reported a history of trauma at the anatomical site were chromoblastomycosis was recorded. The most frequent location of chromoblastomycosis lesions was at the lower limbs, especially on the legs and feet, with a predominance of verrucous and cicatricial lesions. Two patients had mixed lesions (Table 1). Three patients had a lesion in a single anatomical site. In 5 patients, the disease was classified as severe. The most frequently reported symptoms were pruritus (5 patients) and pain (4 patients). One patient presented both symptoms simultaneously. The only reported complication was bacterial infection (4 patients), but none of the patients had a culture‐based diagnosis for this, and the coinfections were treated empirically.

The most frequent comorbidities found were systemic arterial hypertension (6 patients), type II diabetes mellitus (4 patients) and leprosy (3 patients) (Table 1). Five patients received itraconazole treatment combined with cryotherapy and three itraconazole monotherapy. Patients 3 and 10 did not receive antifungal treatment due to their comorbidities. All patients who received antifungal treatment showed improvement in chromoblastomycosis lesions, except patient 6, who had long‐standing chromoblastomycosis.

**FIGURE 2:**
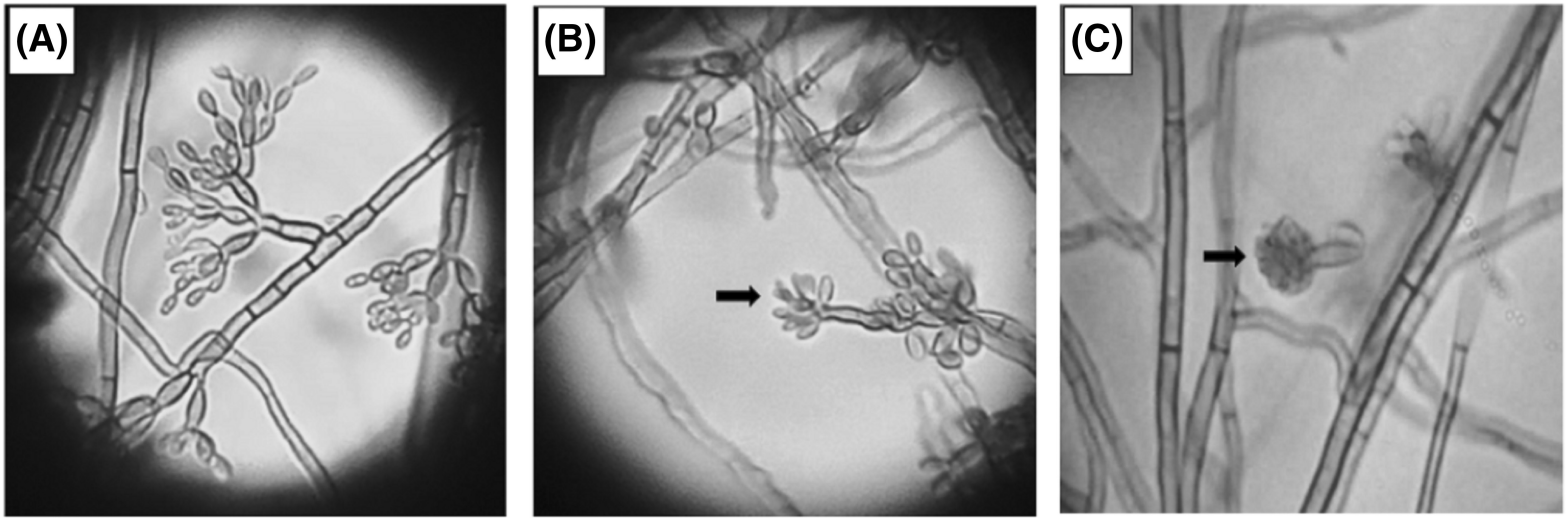
Micromorphological characteristics of *F. pedrosoi* isolates. A: *Cladosporium*‐type sporulation. B: *Rhinocladiella*‐type sporulation. C: *Phialophora*‐type sporulation

**FIGURE 3:**
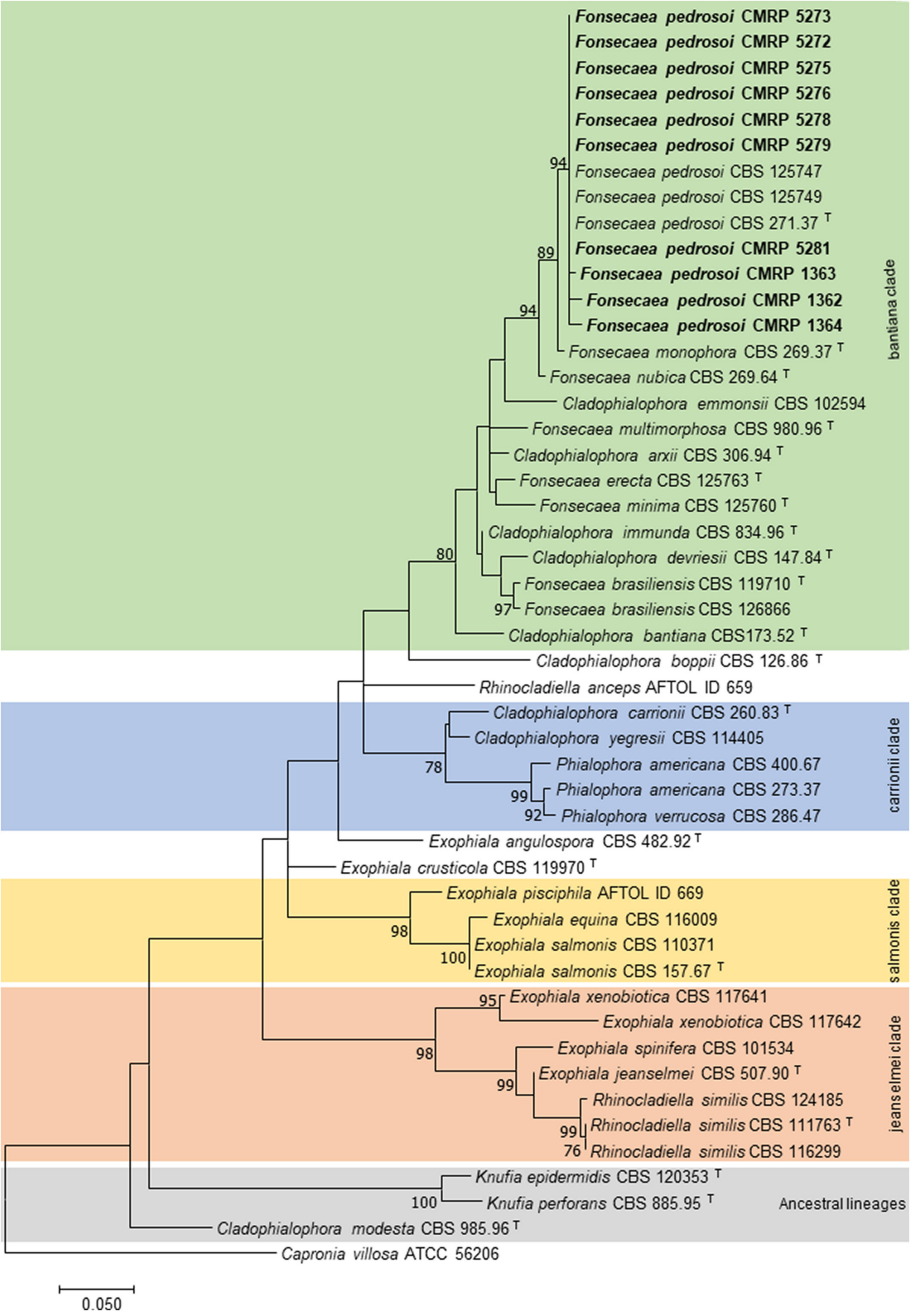
Phylogenetic tree of *F. pedrosoi* isolated from human subcutaneous infection based on combined Internal Transcribed Spacer (ITS) β‐tubulin (*BT2*) sequences. The dendrogram was constructed using maximum likelihood implemented in MEGA v7.0.26 using Tamura‐Nei with gamma invariable sites (TN93 þ G þ I) model. Bootstrap values above 80 from 1000 resampled datasets are shown along the branches. The sequences of the clinical isolates evaluated in this study are indicated in bold. *Capronia villosa* ATCC 56206 (=CBS 616.96) was taken as the outgroup.

Samples were collected from all patients for laboratory studies. Histopathological studies were carried out in 8 patients. Direct mycological examination (KOH 10%) was positive in 8/10 patients and histopathological examination in 5/9 patients. Based on micromorphological characteristics, ten isolates were identified as *Fonsecaea* species. All isolates were molecularly identified as *F. pedrosoi* by sequence analysis of the ITS and partial *BT2* loci (Table 2; Figures 2 and 3).

**TABLE 2:** Histopathology, direct examination, identification of isolates, susceptibility to antifungal agents and ITS sequence data

Patient	Histopathology	Direct examination	Strain	Isolate number	MIC (μg/ml)					GenBank accession no. ITS
					AMB	VCZ	ITZ	5‐FC	TRB	
1	Positive	Positive	*Fonsecaea pedrosoi*	CMRP1362	4	1	0.5	>64	0.5	KX434704
2	Negative	Positive	*Fonsecaea pedrosoi*	CMRP1363	4	0.125	0.5	>64	0.06	KX434705
3	Positive	Negative	*Fonsecaea pedrosoi*	CMRP1364	8	0.25	0.5	>64	0.06	KX434706
4	Negative	Positive	*Fonsecaea pedrosoi*	CMRP5272	8	0.06	0.5	32	0.03	OK183537
5	Negative	Positive	*Fonsecaea pedrosoi*	CMRP5278	4	0.125	0.5	>64	0.125	OK157451
6	Positive	Negative	*Fonsecaea pedrosoi*	CMRP5279	4	0.125	0.25	32	0.125	OK157452
7	Unrealized	Positive	*Fonsecaea pedrosoi*	CMRP5273	4	0.125	0.5	32	0.06	OL677357
8	Positive	Positive	*Fonsecaea pedrosoi*	CMRP5275	4	0.06	0.25	>64	0.06	OK183539
9	Positive	Positive	*Fonsecaea pedrosoi*	CMRP5276	4	0.125	0.25	64	0.125	OK183540
10	Unrealized	Positive	*Fonsecaea pedrosoi*	CMRP5281	4	0.125	0.06	64	0.06	OK157453

Abbreviations: 5‐FC, 5‐fluorocytosine; AMB, amphotericin B; ITZ, itraconazole; MIC, minimal inhibitory concentration, determined by the EUCAST broth microdilution method, definitive document E.DEF 9.3.2(37); TRB, terbinafine; VCZ, voriconazole.

**FIGURE 4:**
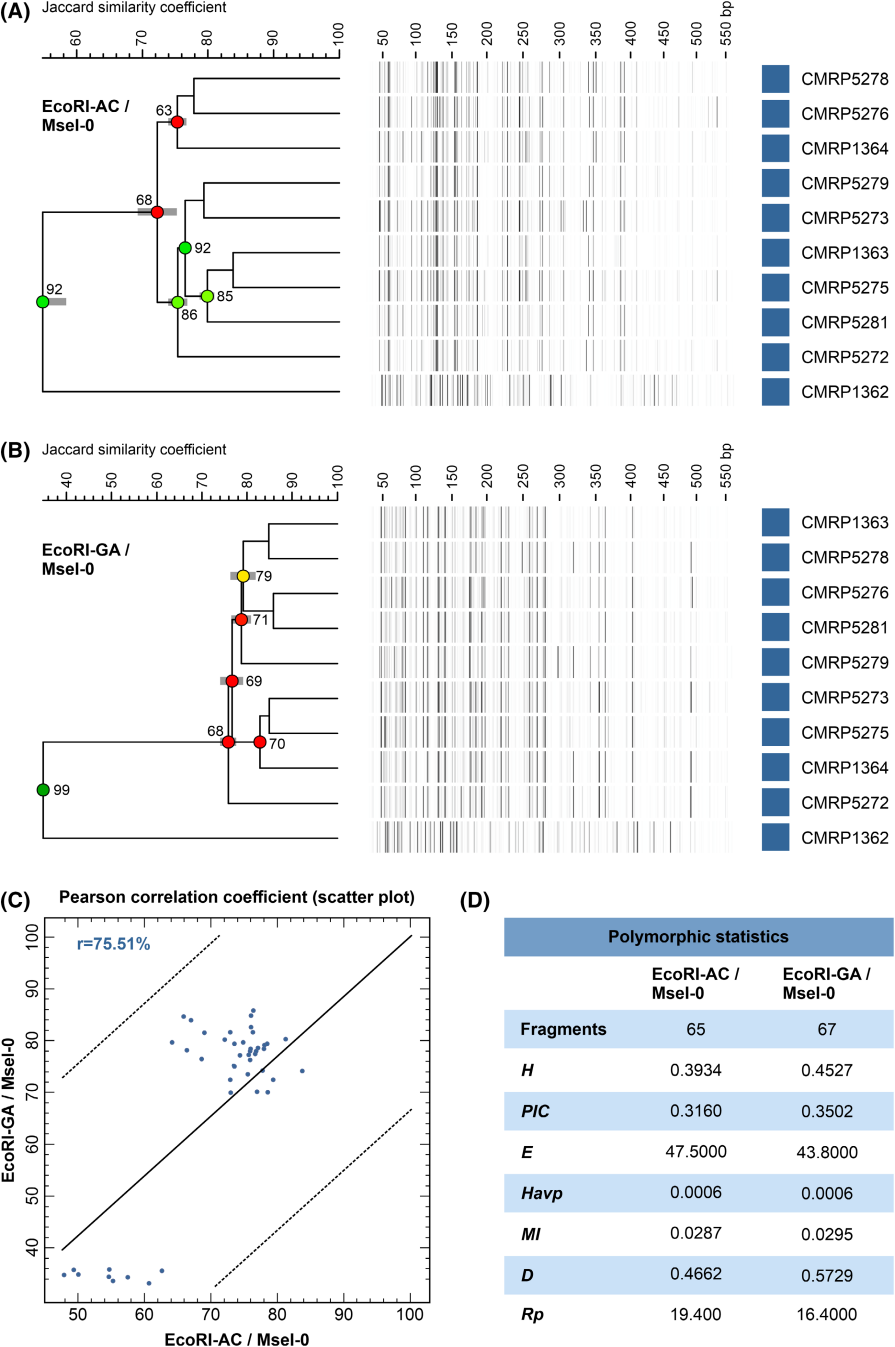
Dendrograms of AFLP fingerprint combinations (A) #1 EcoRI‐AC/MseI‐0, and (B) #2 EcoRI‐GA/MseI‐0. The dendrograms show the clustering profile of the 10 clinical samples of *Fonsecaea pedrosoi*. (C) The correlation between AFLP experiments for combination #1 vs. combination #2 reveals that dendrograms are more congruent than expected by chance (*r* = 75.51%). (D) Polymorphic statistics were calculated for two combinations of markers for *F. pedrosoi*. *D*, discriminating power; *E*, effective multiplex ratio; *H*, expected heterozygosity; *Havp*, mean heterozygosity; *MI*, marker index; *PIC*, polymorphism information content; *Rp*, resolving power.

AFLP fingerprints revealed 65 and 67 fragments in the 50–500 bp range for combinations #1 EcoRI‐AC/MseI‐0 and #2 EcoRI‐GA/MseI‐0, respectively. Cluster analysis and dimensionality reduction methods were useful for determining genetic relationships among all *Fonsecaea* isolates. Typical AFLP dendrograms based on Jaccard's similarity coefficient for *Fonsecaea* isolates are shown in Figure 4. Clustering analysis indicates a moderate level of genetic diversity with a significant global cophenetic correlation coefficient (92%–99%) for both markers supporting a great degree of confidence in the association obtained. This clustering pattern agrees with the generally applied ITS and *BT2*‐based phylogenies. Remarkably, isolate CMRP1362 was distant under both markers (Figure 4A,B), although only a discrete branch was observed in the concatenated phylogenetic analysis. The dendrograms for combinations #1 EcoRI‐AC/MseI‐0 and #2 EcoRI‐GA/MseI‐0 were more congruent than expected by chance, as revealed by the Pearson product–moment correlation coefficient (75.51%) (Figure 4C). The summary of polymorphism indices calculated for selective primers shows a good performance of our AFLP scheme, including good discriminating power (*D* = 0.4662–0.5729) and marker index (*MI* = 0.0287–0.0295), which revealed moderate levels of genetic diversity among *F. pedrosoi* isolates (*PIC* = 0.3160–0.3502, *H* = 0.3934–0.4527 and *Havp* = 0.0006) (Figure 4D).

**FIGURE 5:**
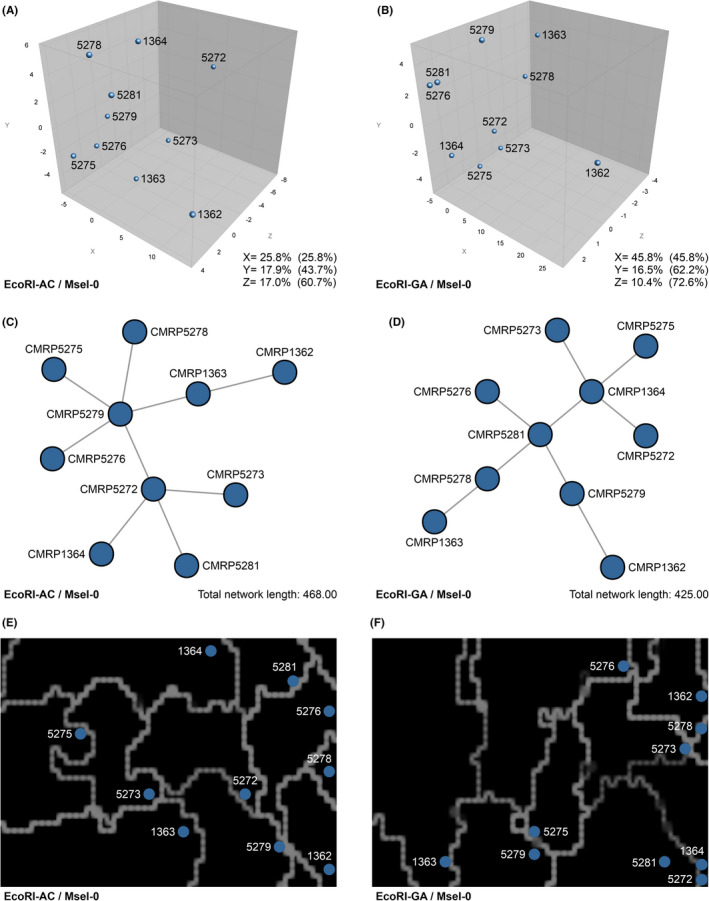
Principal component analysis (PCA) showing the genetic relationship between *Fonsecaea pedrosoi* isolates. The genetic relationship between *F. pedrosoi* isolates is visualised by using AFLP fingerprint data of (A) #1 EcoRI‐AC/MseI‐0 (65 loci), and (B) #2 EcoRI‐GA/MseI‐0 (67 loci). Minimum Spanning Trees (MSTs) using combinations (C) #1 EcoRI‐AC/MseI‐0 (Total network length = 468.00), and (D) #2 EcoRI‐GA/MseI‐0 (Total network length = 425.00). The distribution of the studied AFLP genotypes of 10 *Fonsecaea* isolates originated in the Mato Grosso state, using self‐organising mapping (SOM) for combination (E) #1 EcoRI‐AC/MseI‐0 (65 loci), and (F) #2 EcoRI‐GA/MseI‐0 (67 loci).

Figure 5 illustrates the PCA, MSTs and SOMs plots for both combinations, and the distribution of 10 *Fonsecaea* isolates revealed a similar trend to cluster. Combination #2 EcoRI‐GA/MseI‐0 revealed the highest cumulative percentage explained, with 72.6% of the variation described by the first three components (coordinates X, Y and Z), supporting a robust genetic structure (Figure 5B). The AFLP‐derived MSTs confirmed the genetic structure of *Fonsecaea* isolates, with most isolates having a unique genotype. Isolate CMRP1362 was more randomly distributed in the MSTs and PCA analysis. Finally, 2‐dimensional genetic distance maps (SOMs) revealed that most samples were separated by faint lines, indicating moderate to low diversification. On the contrary, isolate CMRP1362 was separated by bright solid white lines, indicating a more robust genetic distance from the remaining strains (Figure 5E,F).

The antifungal susceptibility profile is shown in Tables 2 and 3. Terbinafine was the most active drug against *F. pedrosoi* isolates, followed by voriconazole and itraconazole. The least active drugs against all isolates were amphotericin B and 5‐flucytosine. The MIC values of the isolates from patients 7, 8 and 9, who had a previous diagnosis of chromoblastomycosis, and the patient with a previous diagnosis of paracoccidioidomycosis and chromoblastomycosis, were similar to the MIC values of the isolates from the patients without earlier treatment.

**TABLE 3:** Geometric mean, rank, MIC_50_ and MIC_90_ of the 10 isolates of *Fonsecaea pedrosoi*

Antifungal	MIC (μg/ml)			
	Geometric mean	Rank	MIC_50_	MIC_90_
Amphotericin B	4.8	4–8	4	8
Voriconazole	0.2	0.06–1	0.125	0.25
Itraconazole	0.381	0.06–0.5	0.5	0.5
5‐Flucytosine	60.8	32 to >64	>64	>64
Terbinafine	0.1205	0.03–0.5	0.06	0.125

Abbreviations: MIC, Minimal inhibitory concentration; MIC_50_ and MIC_90_, Minimal concentration of the antifungal that inhibits the growth of 50 and 90% of the isolates, respectively.

## 4 DISCUSSION

Chromoblastomycosis is a neglected disease in almost all countries and territories of Latin America and the Caribbean. In Brazil, it has been reported in all regions; even so, the prevalence of the disease is unknown because it is not a notifiable disease.

The age of patients with chromoblastomycosis varies in published studies, depending on the population studied. Previous research in Brazil reported cases of chromoblastomycosis in the age group from 7 to 91 years,^7^, ^14^, ^15^, ^17^, ^18^, ^38^, ^39^ but cases from 2 to 99 years of age have been reported from other countries.^40^, ^41^, ^42^, ^43^, ^44^, ^45^ The most affected age group corresponds to the productive age, with a progressive increase in cases from 16 to 20 years old.^41^, ^45^, ^46^, ^47^, ^48^, ^49^, ^50^, ^51^, ^52^ Surveys from China, India and Taiwan reported mean ages of 43.3–65.9 years,^50^, ^53^, ^54^ while other Latin American countries have reports of mean ages from 34 years^55^ to 78 years.^56^ In Brazil, the reported mean age varies between 44.5 and 65.6 years.^7^, ^11^, ^14^, ^15^, ^17^, ^18^, ^20^, ^21^, ^38^, ^39^, ^57^, ^58^, ^59^, ^60^ However, in Brazil, only three studies reported a mean age of 60 years or more,^17^, ^20^, ^38^ similar to the findings of this study.

Chromoblastomycosis was more frequent in males and rural workers, similar to reports from other countries^11^, ^41^, ^44^, ^45^, ^47^, ^49^, ^50^, ^51^, ^52^, ^53^, ^54^, ^55^, ^61^ and Brazil.^7^, ^14^, ^15^, ^17^, ^18^, ^38^, ^57^, ^58^, ^59^, ^60^ Different researchers have postulated that this distribution is related to the fact that men are mainly involved in agriculture, cattle raising and other occupations that expose them to the environments where chromoblastomycosis aetiological agents are present.^7^, ^44^, ^45^, ^47^, ^49^, ^50^, ^51^ Nevertheless, some researchers postulated the possibility that female hormones play a protective role against chromoblastomycosis as has been observed for paracoccidioidomycosis.^4^, ^53^, ^62^ However, two case series from Japan, with 508 patients (1955–2001), a similar proportion of male and female cases was described and thus pleas against a protective role for female hormones.^42^, ^46^

The course of chromoblastomycosis is usually prolonged. In the early stages, the disease is asymptomatic or oligosymptomatic. Thus, patients do not seek medical help, leading to delayed diagnosis and disease progression.^4^, ^5^, ^9^ The mean evolution time of chromoblastomycosis found in this study was 8.6 years; however, in most case series published in Latin America, including Brazil, the mean evolution time was 10 or more years and ranged from 1 month to 40 years.^7^, ^15^, ^17^, ^18^, ^20^, ^21^, ^38^, ^39^, ^45^, ^47^, ^49^, ^57^, ^58^, ^59^, ^60^, ^63^, ^64^, ^65^, ^66^, ^67^ Research carried out in Brazil,^20^ Mexico^45^ and Venezuela^55^ described patients aged 50 and 74 with many years of progressive disease evolution.

Lesions associated with chromoblastomycosis usually start at the site of inoculation of the aetiologic agent, usually in unprotected areas of the body, being more frequent in the lower limbs followed by the upper limbs, as found in this study. This location of the lesions is related to activities in rural areas in developing countries, such as Brazil, where workers generally do not wear adequate protective clothing on the lower limbs and are exposed to injuries caused by wood, thorns and stones, facilitating the implantation of the fungus in tissues^7^, ^11^, ^14^, ^17^, ^21^, ^38^, ^45^, ^48^, ^49^, ^50^, ^51^, ^52^, ^53^, ^57^, ^60^, ^68^ but in research carried out in Venezuela,^41^, ^43^, ^47^, ^55^ Cuba,^69^ Japan,^42^, ^46^ Taiwan^54^ and Australia,^44^, ^70^ the upper limbs were the most affected.

The lesions mainly described were of the verrucous type, coinciding with studies published in Brazil and other countries.^7^, ^11^, ^15^, ^17^, ^18^, ^21^, ^52^, ^54^, ^57^, ^64^, ^66^, ^71^, ^72^, ^73^, ^74^ Nevertheless, patients with chromoblastomycosis of chronic evolution may present different types of lesions simultaneously.^7^

Itching and pain were the main symptoms reported. Few studies described patients' symptoms, and most reported these same symptoms.^7^, ^11^, ^15^, ^46^, ^52^, ^59^, ^60^, ^62^, ^72^

The complications mainly described in chromoblastomycosis have been secondary bacterial infections and carcinomatous degeneration of the lesions.^7^, ^11^, ^15^, ^17^, ^45^, ^50^, ^52^, ^57^, ^62^, ^71^, ^75^, ^76^ The complication found in patients from Mato Grosso was a bacterial infection. This complication is mainly caused by Gram‐positive cocci in the upper limbs and Gram‐negative bacilli in the lower limbs. *Staphylococcus aureus* is the commonly involved microorganism in secondary infections, and mixed infection with *Streptococcus pyogenes* is commonly associated with severe lesions.^76^

Three patients had chromoblastomycosis‐leprosy coinfection. There are few reports of this coinfection, most of them from Brazil.^7^, ^17^, ^46^, ^50^, ^77^, ^78^, ^79^, ^80^, ^81^ Two of the patients reported in this research were elderly (65 years or older), coinciding with two previous reports.^77^, ^79^ The explanation for the occurrence of chromoblastomycosis‐leprosy coinfection in the elderly can be very complex and could be related to immunosenescence.

The treatment of chromoblastomycosis is difficult, and it is usual to observe little success in the treatment and frequent relapses, mainly due to the diversity of aetiological agents, their susceptibility to available antifungals, pharmacokinetics and pharmacodynamics of antifungals and the duration and extent of lesions.^5^, ^50^, ^53^ The most common causes of treatment failure include treatment abandonment, prolonged therapy, side effects and antifungal resistance.^50^, ^51^, ^53^, ^54^ However, the severity of the lesions, the time of disease evolution and the individual response to treatment also influence the final result.^82^, ^83^ This is also the reality for patients in Mato Grosso: prolonged evolution of the disease, treatment abandonment, multiple treatments with the improvement of the lesions without a clinical cure or any success.

As reported by other studies, the genus *Fonsecaea* is globally the main aetiologic agent of chromoblastomycosis, but there are some differences in its geographic distribution. *F. pedrosoi* is the predominant species in Latin America while *F. monophora* and *F. nubica* are the main agents of chromoblastomycosis in Asia.^11^, ^52^, ^84^ In the case series published in Brazil up to 2010, *F. pedrosoi* was the most frequent aetiologic agent of chromoblastomycosis, followed by *C. carrionii* and *Phialophora verrucosa*,^14^, ^15^, ^18^, ^38^, ^57^, ^58^ all identified by the micromorphology of the fungus. In more recent publications, using molecular techniques for species identification, they found that *F. pedrosoi* is the most frequent species in Brazil, followed by *F. monophora*, *F nubica* and *F. pugnacius*.^7^, ^17^, ^21^, ^23^, ^39^ Still, there are case reports produced by *C. carrionii*,^17^, ^85^, ^86^ *Rhinocladiella aquaspersa*,^87^, ^88^, ^89^ *R. similis*,^21^ *R. tropicalis* ^7^ and *Cyphellophora ludovicensis* ^7^ most of them identified by molecular techniques.

Judging from the AFLP analysis, we report moderate genetic diversity, which is notable given the small set of samples evaluated here. Remarkably, we observed many invariant fragments detected among *Fonsecaea* isolates collected at a close geographic distance from each other in the Mato Grosso state. This genetic proximity agrees with the finding reported by Najafzadeh et al^90^ and suggests that vectors of dispersal for *Fonsecaea* species are slow, leading to detectable regional diversification. A similar conclusion was drawn recently for members of the *Paracoccidioides brasiliensis* complex showing a narrow geographical range, such as *Paracoccidioides venezuelensis* and *Paracoccidioides restrepiensis*.^30^

The antifungal susceptibility profile of *F. pedrosoi* isolates was similar to those reported in other studies, regardless of the susceptibility method used.^39^, ^91^, ^92^, ^93^ However, a survey carried out in the Rondônia state, Brazil, reported lower MIC values for eight *F. pedrosoi* isolates, using the same antifungal agents and methodology.^21^

There are no cut‐off values to define antifungal sensitivity or resistance for the aetiologic agents of chromoblastomycosis. Still, some authors consider that MIC values ≤1 μg/ml could indicate sensitivity for most antifungal drugs used to treat diseases caused by melanized fungi.^94^ Considering this value, it could be interpreted that the tested isolates are susceptible to itraconazole, voriconazole and terbinafine, but these values must be carefully interpreted because there are often discrepancies between the results of the susceptibility tests and the clinical outcomes.^4^, ^93^ These three antifungals, especially itraconazole and terbinafine, are the best currently available alternatives for the treatment of chromoblastomycosis, with a variable success rate.^4^, ^5^

The low number of patients and isolates included in this study represents a limitation of this study, as well as the difficulty in obtaining data about the treatment and therapeutic response of patients diagnosed and treated previously in other healthcare centres, but it confirms the presence of chromoblastomycosis in the Mato Grosso state, Brazil, and reveals this neglected disease as a possible public health problem that needs the attention of health authorities.

According to the data we presented here, chromoblastomycosis is present in Mato Grosso state, Brazil, affecting people in rural and urban areas, causing disability and personal, family and economic damage. Knowledge of the epidemiology of this disease, its main aetiologic agent and the confirmation of itraconazole, voriconazole and terbinafine as the best therapeutic alternatives, offer a regional perspective on this neglected disease. This initial characterisation of chromoblastomycosis in central western Brazil can serve as a basis for the formulation of educational programs aimed at the community and health professionals. Such education needs to cover preventive actions, early diagnosis, immediate and adequate treatment, as well as encouraging the creation of a system of epidemiological surveillance, which includes the compulsory notification of cases, the active search for them, the molecular identification of the aetiological agents, the formulation of clinical studies and new treatments, ultimately reducing the costs for society and the Brazilian health system.

## AUTHOR CONTRIBUTIONS

Armando Guevara: Writing—original draft; Investigation; Formal analysis; Writing—review & editing. Andréia Ferreira Nery: Investigation; Formal analysis; Writing—review & editing; Writing—original draft. Márcia de Souza Carvalho Melhem: Investigation; Formal analysis; Writing—review & editing. Lucas Bonfietti: Investigation; Formal analysis; Writing—review & editing. Anderson Messias Rodrigues: Conceptualization; Investigation; Funding acquisition; Writing—original draft; Visualization; Writing—review & editing; Methodology; Validation; Software; Formal analysis; Project administration; Data curation; Resources. Ferry Hagen: Writing—review & editing; Methodology; Validation; Formal analysis. Jamile Ambrósio de Carvalho: Investigation; Formal analysis; Writing—review & editing. Zoilo Pires de Camargo: Writing—original draft; Writing—review & editing; Investigation; Formal analysis; Supervision. Bruna Jacomel F. de Souza Lima: Investigation; Formal analysis; Writing—review & editing. Vânia Aparecida Vicente: Writing—original draft; Funding acquisition; Investigation; Conceptualization; Methodology; Visualization; Writing—review & editing; Validation; Software; Formal analysis; Project administration; Resources; Supervision; Data curation. Rosane Christine Hahn: Conceptualization; Investigation; Writing—original draft; Funding acquisition; Writing—review & editing; Visualization; Validation; Methodology; Software; Formal analysis; Project administration; Data curation; Supervision; Resources.

## CONFLICT OF INTERESTS

The authors declare that there are no conflicts of interest.

## Data Availability

The data that support the findings of this study are openly available in NCBI Genbank at https://www.ncbi.nlm.nih.gov/. Accession numbers are provided in Table 2.
